# Euglycemic Diabetic Ketoacidosis in a Young Pregnant Woman Precipitated by Urinary Tract Infection

**DOI:** 10.7759/cureus.7331

**Published:** 2020-03-19

**Authors:** Vijayadershan Muppidi, Sreenath Meegada, Tejo Challa, Suman Siddamreddy, Subhankar Samal

**Affiliations:** 1 Internal Medicine, Indiana University Health, Indianapolis, USA; 2 Internal Medicine, The University of Texas Health Science Center/Christus Good Shepherd Medical Center, Longview, USA; 3 Internal Medicine, Baptist Health Medical Center, North Little Rock, USA; 4 Internal Medicine, Ascension Health, Milwaukee, USA

**Keywords:** euglycemic dka, diabetic ketoacidosis, pregnancy and dka, urinary tract infection dka

## Abstract

Diabetic ketoacidosis (DKA) is a life-threatening diabetic complication and medical emergency. Euglycemic DKA (EKDA) is a variant of DKA with normal range glucose levels. The condition can be difficult to diagnose due to the misleading euglycemic levels. Pregnancy, infection, low-calorie intake, and use of insulin are some of the common etiologies of EDKA. We report a case of a young, pregnant female, with type I diabetes mellitus, in her third trimester admitted with EKDA. The EKDA was triggered by urinary tract infection (UTI), and the patient had other etiologies that have predisposed her to EKDA. Along with the case presentation, we discuss the common etiologies, pathophysiology, and management of EKDA. Euglycemic DKA is a life-threatening emergency that needs to be recognized early and treated aggressively, especially in pregnant patients, to avoid deleterious effects to maternal and fetal health.

## Introduction

Diabetic ketoacidosis (DKA) is a well known and potentially life-threatening clinical condition. The hallmark of DKA is hyperglycemia (serum glucose > 250 mg/dL), ketosis (presence of excess ketone bodies in blood), and anion gap metabolic acidosis (arterial PH<7.3 and serum bicarbonate <18 meq/L) [1]. Euglycemic DKA (EDKA) is defined as an entity of DKA with metabolic acidosis, ketosis, but with glucose levels less than 200 mg/dL [2]. It is not as common as DKA and is relatively harder to diagnose due to the misleading normal range of blood glucose levels. Euglycemic DKA was first reported by Munro et al. in 1973. In the case series on DKA by Munro et al., about 17.5% of patients had EDKA [3]. The condition seems to be more common than usually perceived to be by the medical community. Pregnancy and urinary tract infection (UTI) are triggers for EDKA [4]. We present a case of a young, pregnant female with diabetes mellitus type I with UTI who presented with EDKA. We discuss the etiology, pathophysiology, and management of EDKA.

## Case presentation

A 23-year-old pregnant female with a history of type I diabetes mellitus presented to the emergency room (ER) with a two-day history of nausea and vomiting. She was 37 weeks pregnant at the time. Vomitus was non-bilious and non-bloody in nature. She checks blood glucose at home and reported glucose levels in the range of 130 to 170 mg/dL. She denied abdominal pain, no food poisoning, no eating out, she has been eating normally until these symptoms started. Upon further questioning, she denied fevers, chills, rigors, and sick contacts. There was no change in appetite or weight. She denied urinary symptoms. She was on intermediate-acting insulin (isophane) twice a day, and short-acting insulin (Humalog®) with carbohydrate correction regimen before each meal.

On initial evaluation in the ER, she was dehydrated. She was in mild distress from nausea and vomiting. Vital signs were significant for tachycardia with a heart rate of 110 bpm, tachypnea with a rate of 24/minute, normal range blood pressure, and oxygen saturation. The respiratory exam was significant for tachypnea, the cardiovascular exam was significant for tachycardia, the abdomen exam was benign, and the fetus's heart sounds were normal.

Labs showed (Table 1) normal complete blood cell count; chemistry panel showed low sodium of 133 meq/L, bicarbonate of 16 meq/L, anion gap of 13 meq/L, and elevated ketones in the blood. Urine analysis was done in a clinic that showed elevated glucose and elevated white cell count of 10/high power field, and serum lactic acid was normal at 1.5 millimoles/liter, and then she was referred to the emergency room for further evaluation considering her third-trimester pregnancy. The patient was started on high dose sliding scale insulin and empiric ceftriaxone and was admitted to the obstetric floor. Subsequent lab workup showed a worsening anion gap of 18, bicarbonate of 6 meq/L, and sodium of 130 meq/L (Table 2).

**Table 1:** Basic metabolic panel showing euglycemic diabetic ketoacidosis

Laboratory findings	
Sodium	133
Potassium	4.3
Chloride	104
Carbondioxide	16
Anion gap	13
Blood urea nitrogen	6
Creatinine	0.56
Glucose	187
Albumin	2.8

**Table 2:** Subsequent basic metabolic panel showing worsening euglycemic diabetic ketoacidosis

Laboratory findings	
Sodium	130
Potassium	3.6
Chloride	106
Carbondioxide	6
Anion gap	18
Creatinine	0.76
Glucose	111

Urine culture was positive for Escherichia coli. Internal medicine consultation was sought at this point. A diagnosis of euglycemic type I diabetic ketoacidosis (DKA) precipitated by UTI was made. She was started on insulin drip along with dextrose containing intravenous fluids. She started feeling better 24 hours after being on insulin drip with improvement in her nausea and vomiting. Repeat labs showed normal anion gap and normal bicarbonate (Table 3).

**Table 3:** Repeat basic metabolic panel (after treatment with intravenous insulin and fluids) showing normalization of anion gap

Laboratory findings	
Sodium	137
Potassium	3.2
Chloride	107
Carbon dioxide	22
Anion gap	8
Blood urea nitrogen	<3
Creatinine	0.46
Glucose	119

The patient started tolerating oral food and was transitioned to subcutaneous insulin per her home regimen.

## Discussion

Hyperglycemic emergencies in diabetics include DKA and hyperglycemic hyperosmolar state (HHS). EDKA is a variant of DKA with normal range glucose levels. Diagnosis of EDKA can be difficult due to the misleading normoglycemia. Other causes of ketoacidosis (such as alcoholic ketoacidosis and starvation ketoacidosis) and causes of increased anion gap acidosis (methanol, uremia, paraldehyde, lactic acidosis, ethylene glycol, and salicylate) need to be ruled out. DKA is caused by insulin deficiency and the rise of counter-regulatory hormones (such as glucagon, growth hormone, cortisol, catecholamines) on the contrary. Both insulin deficiency and an increase in counter-regulatory hormones lead to decreased glucose utilization by peripheral tissues and an increase in substrates for gluconeogenesis; both contribute to hyperglycemia. In addition, increased glycogenolysis (again by the rising counter-regulatory hormones) contributes to hyperglycemia as well. Hyperglycemia leads to glucosuria, loss of water, dehydration, and subsequently, renal dysfunction. Insulin deficiency causes increased lipolysis and generation of keto acids in the liver, which in turn leads to metabolic acidosis and ketosis [5]. The metabolic effects and complications are more or less the same in EKDA as well.

DKA is more common in type I as compared to type II diabetes mellitus [6]. The degree of glycemia differentiates euglycemic DKA from classical DKA. Munro et al. (1973) and later, Jenkins et al. (1993) described EKDA with glucose levels of less than 300 mg/dL [3, 7]. Currently, EKDA is recognized as a form of DKA with serum glucose levels of less than 200 mg/dL. Based on the cut off of less than 200 mg/dL, the incidence of EDKA among studies by Munro et al. and Jenkins et al. is 7.5% (16/211 DKA episodes) and 0.8% (6/722 DKA episodes). Patients in DKA that are still administering some amount of insulin present with lower glucose levels but still have ketosis and acidosis and are diagnosed with EKDA [8]. In addition, decreased glucose production due to fasting state or decreased calorie intake contributes to lower glucose levels as well in EDKA. Common triggers associated with EDKA include starvation, dehydration, pregnancy, sodium glucose co-transporter 2 (SGLT2) inhibitors, glycogen storage disorders, and chronic liver disease, alcohol intoxication, sepsis, and cocaine intoxication [4, 8, 9]. 

Jaber et al. reported a case of a young pregnant female with EKDA with fetal demise. In the report, the authors also summarized 20 previously reported cases of EKDA in pregnancy; most of them were in the third trimester [10]. Since then, a few more case of EDKA in pregnancy has been reported. Previously, there was one case report of EDKA in a diabetic (type I), pregnant female in the third trimester triggered by UTI [11]. Our case is only the second instance of a pregnant female in the third trimester with EKDA triggered by UTI/sepsis.

We present a case of a young pregnant female in her third trimester of pregnancy who presented with euglycemic DKA. The patient did meet all the criteria for euglycemic DKA, including glucose less than 200 mg/dL, metabolic acidosis, and ketosis. The index patient was predisposed to EDKA due to underlying type I diabetes mellitus, pregnancy, and use of insulin. The episode of UTI and associated nausea, vomiting, and low-calorie intake triggered the EKDA. Mechanisms of glycemic control change with each trimester of pregnancy. In the first trimester, placental steroids lead to increased insulin production and decreased fasting glycemia [12]. In later stages of pregnancy, there is an increase in counterregulatory hormones (such as human placental lactogen [HPL]) and decrease in insulin deficiency due to hormonal changes (increase in estrogens, progesterone, HPL, tumor necrosis factor-alpha) both of which lead to hyperglycemia [13, 14]. In late pregnancy, the dwindling glucose levels due to increased demand from fetus and placenta, decreased glycogenolysis and gluconeogenesis along with increased renal loss of glucose, causes maternal metabolism to enter a catabolic state and switch to fat as a primary fuel for energy [12]. Increased lipolysis and free fatty acid delivery to the liver causes increased ketogenesis. Also, the respiratory alkalosis in late pregnancy causes increased urinary excretion of bicarbonate and metabolic acidosis [15]. Thus, pregnancy leads to euglycemia, ketosis, and acidosis.

The index case also had fasting and decreased calorie intake from nausea and vomiting prior to arrival. This causes increased lipolysis, free fatty acid production, and ketogenesis [16]. The patient continued to take insulin, which likely contributed to normoglycemia but did not suppress lipolysis and ketogenesis [17]. Infection or sepsis is known to trigger DKA in diabetic patients due to an increase in counter-regulatory hormones [5].

EDKA during pregnancy is associated with multiple deleterious effects on fetal and maternal health. Some of the fetal complications include uterine hypoperfusion, fetal hypoxia, recurrent late decelerations and impaired fetal brain development [14, 18, 19]. DKA is also associated with increased maternal mortality. The cause of the acidosis needs to be recognized early and treated aggressively. Emergency Cesarean section is reserved for cases with worsening maternal health as it is associated with significant maternal morbidity and mortality [10]. The index case was treated aggressively and successfully. She had a normal vaginal delivery later, and both mother and baby were healthy at follow up visits.

The treatment of EDKA is similar to DKA except for the need for intravenous fluids with higher glucose concentration due to the lower levels of glucose in EDKA. Due to the life-threatening nature of EDKA, management should adopt an aggressive approach towards the correction of acidosis and volume depletion with large amounts of intravenous fluids, intravenous insulin, and correction of electrolytes [20]. Serial chemistry profile and anion gap closure are important parameters in monitoring the response. While treating ketoacidosis, the underlying cause should be sought and treated as well. In our patient, underlying UTI was diagnosed and effectively treated. The fetal heart should be monitored closely during the management of EDKA.

## Conclusions

Euglycemic DKA is a form of DKA with lower blood glucose levels. The diagnosis can be difficult due to the deceiving lower blood glucose levels as compared to the classical hyperglycemic levels seen in DKA. Pregnancy is now a recognized cause of euglycemic DKA. Early diagnosis and management are very crucial in preventing potential serious metabolic complications and mortality.
